# A dataset of plant and microbial community structure after long-term grazing and mowing in a semiarid steppe

**DOI:** 10.1038/s41597-020-00738-1

**Published:** 2020-11-19

**Authors:** Wenhuai Li, Jasna Hodzic, Jishuai Su, Shuxia Zheng, Yongfei Bai

**Affiliations:** 1 School of Ecology and Environment, Inner Mongolia University, Hohhot, 010021 China; 2 School of Environmental and Forest Sciences, University of Washington, Seattle, Washington 98195 USA; 3 State Key Laboratory of Vegetation and Environmental Change, Institute of Botany, Chinese Academy of Sciences, Beijing, 100093 China; 4 College of Resources and Environment, University of Chinese Academy of Sciences, Beijing, 100049 China

**Keywords:** Grassland ecology, Community ecology, Biodiversity

## Abstract

Grazing and mowing are two dominant management regimes used in grasslands. Although many studies have focused on the effects of grazing intensity on plant community structure, far fewer test how grazing impacts the soil microbial community. Furthermore, the effects of long-term grazing and mowing on plant and microbial community structure are poorly understood. To elucidate how these management regimes affect plant and microbial communities, we collected data from 280 quadrats in a semiarid steppe after 12-year of grazing and mowing treatments. We measured plant species abundance, height, coverage, plant species diversity, microbial biomass, and microbial community composition (G+ and G− bacteria; arbuscular mycorrhizal and saprotrophic fungi; G+/G− and Fungi/Bacteria). In addition, we determined the soil’s physical and chemical properties, including soil hardness, moisture, pH, organic carbon, total nitrogen, and total phosphorus. This is a long-term and multifactorial dataset with plant, soil, and microbial attributes which can be used to answer questions regarding the mechanisms of sustainable grassland management in terms of plant and microbial community structure.

## Abstract (summary)

**Table:** 

Measurement(s)	microbial community • plant trait • Abundance • composition of soil • Microbial DNA • acidity of soil • phosphorus • organic material • plant canopy cover • plant height • humidity of soil • hardness of soil • concentration of carbon atom in soil • concentration of nitrogen atom in soil • fungi phospholipid fatty acids • bacteria phospholipid fatty acids
Technology Type(s)	field work • DNA sequencing • pH meter • ultraviolet spectrometer • chloroform-extraction method • visual observation method • oven drying • soil hardness tester • Walkley-black method • colorimetry • gas chromatography • micro-Kjeldahl digestion
Factor Type(s)	long-term grazing • long-term mowing
Sample Characteristic - Organism	Bacteria • Fungi • Embryophyta
Sample Characteristic - Environment	steppe • grassland area
Sample Characteristic - Location	Inner Mongolia Autonomous Region

Machine-accessible metadata file describing the reported data: 10.6084/m9.figshare.13162052

## Background & Summary

Grasslands cover about 40% of the Earth’s land surface (excluding Greenland and Antarctica)^1^. Livestock grazing in grasslands has resulted in widespread alterations in biodiversity and ecosystem functions and services^2,3^, with responses to livestock being largely dependent on grazing intensity, herbivore type, and site productivity^4–6^. Livestock grazing can affect plant and microbial communities via both direct and indirect pathways^7^. For example, selective consumption of high-quality forage by herbivores can directly structure heterogeneous vegetation^8,9^, while trampling indirectly affects plant and microbial growth by increasing soil compaction and changing soil structure^10,11^. In addition, the deposition of dung and urine by herbivores can redistribute nutrients in soil, indirectly affecting plant and microbial growth^12,13^. Despite an increased knowledge of how grazing can change aboveground plant species diversity and composition, relatively little is known about how grazing influences belowground soil microbial community structure.

Mowing for hay is another common practice in grassland management which profoundly affects plant and microbial communities in different ways^14,15^. Unlike grazing, mowing does not selectively impact palatable plants and does not cause trampling or dung and urine deposition. Furthermore, although it has less of an impact on soil structure than grazing, mowing can produce a more uniform vegetative cover and remove plant aboveground biomass, reducing nutrient cycling to the soil^16^. However, due to the cumulative nature of mowing and grazing impacts, the effects on the plant and microbial community may only be significant after long-term exposure.

In 2005, a long-term grassland management experiment was established in a semiarid steppe of China. It was used to compare how different grazing intensities (0, 1.5, 3.0, 4.5, 6.0, 7.5, 9.0 sheep ha^−1^) and management regimes (enclosure, grazing, mowing) affect grassland biodiversity and ecosystem functioning. After 12 years of management, we measured a suite of indices. For each of the 280 quadrats, we measured plant community composition, plant height and cover, microbial community composition and biomass, and the physical and chemical properties of the soil. This dataset can be used to (i) assess how plant and microbial community structure changes with increasing grazing intensity; (ii) decide the suitable grazing intensity to maintain community composition; (iii) demonstrate changes to plant-soil-microbe interactions under grazing and mowing regimes; (iv) determine whether a grazing or mowing regime is more suitable for long-term management in a semiarid steppe. The results from the analysis of this dataset can provide useful recommendations for sustainable grassland management.

## Methods

### Site description

The study site is a typical semiarid grassland representative of the Eurasian steppe^17^, located in the Xilin River Basin, Inner Mongolia Autonomous Region of China, close to the Inner Mongolia Grassland Ecosystem Research Station (IMGERS, 43°38′ N, 116°42′ E). Mean annual precipitation is 346 mm, with 60–80% of precipitation falling in the growing season (May to September). Mean annual temperature is 0.3 °C, with mean monthly temperatures ranging from −21.6 °C in January to 19.0 °C in July^4^. The topography at our experimental site consists of two landscape units (i.e. flat block and sloped block), with elevation ranging from 1200 to 1280 m above sea level, and slopes less than 5°^18,19^. The soil is classified as dark chestnut (Calcic Chernozem, ISSS Working Group RB, 1998) derived from aeolian sediments^18,20^. The soil substrate is dominated by sandy loam and loamy sand with more than 50% being fine sand and silt^21^. At the beginning of the experiment, soil organic carbon and total nitrogen contents were higher in the flat block than in the sloped block (Table 1). Plant species richness and above-ground biomass were also greater in the flat block than in the sloped block, although species composition in terms of relative biomass of common species did not differ between the two systems (Table 1). *Leymus chinensis* (perennial rhizomatous grass) and *Stipa grandis* (perennial bunchgrass) are the dominant species in the study area, together accounting for more than 70% of community aboveground biomass. Other dominant species include *Cleistogenes squarrosa*, *Agropyron cristatum*, *Achnatherum sibiricum*, and *Carex korshinskyi*.

**Table 1:** Soil and vegetation characteristics in the flat and sloped blocks prior to grazing and mowing interventions.

Soil and vegetation properties	Flat block	Sloped blcok
Soil carbon content (g kg^−1^)	19.78 ± 0.73**	17.39 ± 0.49
Soil nitrogen content (g kg^−1^)	1.94 ± 0.08**	1.68 ± 0.04
C: N ratio	10.32 ± 0.17	10.35 ± 0.19
Soil bulk density (g cm^−3^)	1.19 ± 0.02	1.18 ± 0.02
Number of plant species	41	20
Above-ground biomass (g m^−2^)	129.02 ± 15.52*	77.06 ± 7.67
Relative biomass of common species (%)		
*Leymus chinensis*	41.69 ± 5.86	43.18 ± 7.99
*Stipa grandis*	36.22 ± 4.69	35.32 ± 8.64
*Cleistogenes squarrosa*	9.29 ± 1.83	11.32 ± 3.31
*Carex korshinskyi*	5.91 ± 2.43	5.62 ± 1.50
*Agropyron cristatum*	2.24 ± 0.98	2.03 ± 1.21
*Achnatherum sibiricum*	0.30 ± 0.17	0.63 ± 0.38

Significant differences between flat and sloped blocks are reported from one-way ANOVA as *P < 0.05; and **P < 0.01.

### Study design

**Fig. 1:**
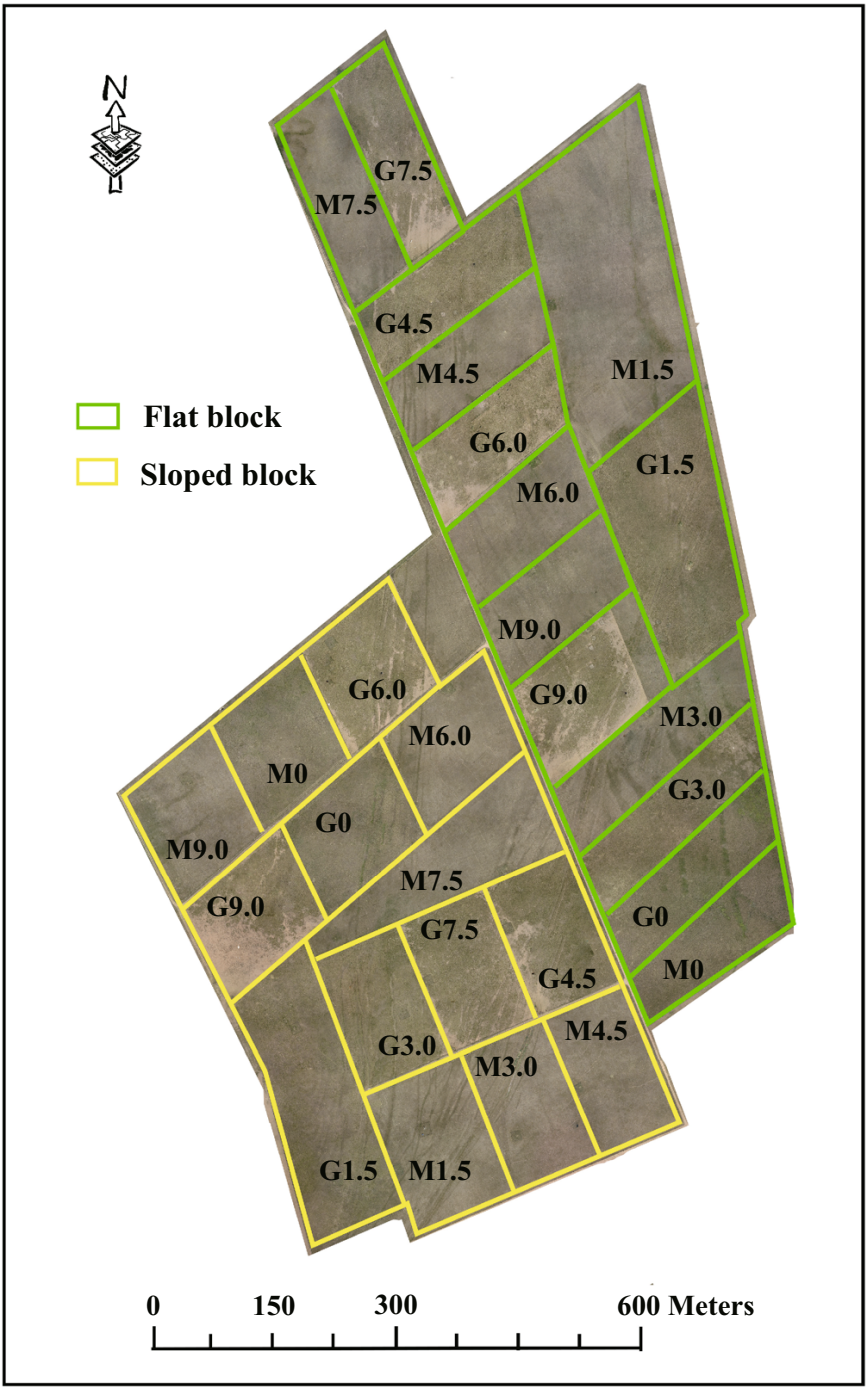
Illustration of the grazing experiment design. G: grazing regime, M: mowing regime.

The experimental area was used for moderate sheep grazing (1.5–3 ewes ha^−1^ year^−1^) by local herdsmen until 2003. Afterwards, grass swards recovered for two years before the experiment started^20,22^. At the end of the growing season in 2004, prior to beginning the experiment, swards in the entire area were cut to 3–5 cm in stubble height^23^. The experiment was established in June 2005 with split plots in a randomized complete block design (Fig. 1). The study area included two blocks (i.e., flat and sloped blocks), with each block further divided into seven plots. We included flat and sloped blocks because our project was designed to assess the impacts of grazing at spatial scales that are both relevant to land management and that can capture ecosystem and landscape-scale effects of grazing^24^. It is unrealistic to conduct such a study in an area with no variation in topography. Grazing intensity was randomly assigned to the plots, and each plot was divided into two subplots. The grazing or mowing management regime was randomly assigned into each subplot^23^. In the grazing regime, there were seven levels of grazing intensity (GI: 0, 1.5, 3.0, 4.5, 6.0, 7.5 and 9.0 sheep ha^−1^), and sheep grazed in the subplots continuously from June to September each year^25^. The ungrazed plots (0 sheep ha^−1^) had no sheep grazing for 12 years. Each subplot was 2 ha, except the subplot with 1.5 sheep ha^−1^, which was enlarged to 4 ha to ensure a minimum herd of six sheep per subplot. In the mowing regime, mowing was done once a year in the middle of August. Plant and soil microbial community data was collected in late July and early August 2017, after 12 years of grazing and mowing treatments.

### Plant community surveys

For each subplot, we randomly laid out ten 1 m × 1 m quadrats at least five meters from the edge of each plot to avoid edge effects. In each quadrat, plant species were identified, and the abundance of each species was counted by bunches (bunchgrasses) or stems (rhizomatous grasses). For each species, five individuals were randomly chosen to measure plant height and the average height of all species was used as plant canopy height. Plant canopy coverage was measured visually.

### Soil sampling

For each quadrat, three soil cores (3 cm diameter, 10 cm depth) were collected, and soil was passed through a 2 mm sieve to form one composite soil sample per quadrat. Sieved soil was then divided into three subsamples. One subsample was air-dried for the analysis of soil pH, soil organic carbon (SOC), total nitrogen (TN), and total phosphorus (TP). The second fresh subsample was used for the analysis of microbial community structure and microbial biomass. The third subsample was stored at -20 °C prior to being used for microbial sequencing analysis.

### Soil physical and chemical properties

To evaluate soil compaction, we measured soil hardness by using a Yamanaka-style soil hardness tester (Fujiwara Scientific Co., Japan). Soil moisture content was measured by using 10 g of moist soil that was oven-dried at 105 °C for 24 h. Soil pH was measured in a 1:2.5 soil:water suspension using a pH meter (FE20-FiveEasy, Mettler-Toledo, Switzerland).

We measured SOC content with the Walkley-black method, soil TN content by the micro-Kjeldahl digestion, followed by colorimetric determination with a 2300 Kjeltec Analyzer Unit, and soil TP content was by the H_2_SO_4_-HClO_4_ fusion method using a 6505 UV spectrophotometer^26^.

### Soil microbial community structure

Microbial community structure was assessed using phospholipid fatty acids (PLFAs), as described by Bossio and Scow^27^. First, lipids were extracted from 10 g of fresh soil using a buffer (CHCL_3_:CH_3_OH:K_2_HPO_4_ = 1:2:0.8, v:v:v). Second, the fatty acid methyl esters (FAMEs) were separated, quantified and identified using a gas chromatograph system (Agilent 7890, Santa Clara, USA) and a MIDI Sherlock Microbial Identification System (MIDI Inc., Newark, USA). Peak areas were converted to nmol g^−1^ dry soil using the internal standard, methylnon-adecanoate (C19:0). Third, the specific microbial groups were identified according to their representative markers. Specifically, G+ bacteria correspond to iso-, anteiso- and 10Me-branched PLFAs; G- bacteria correspond to monounsaturated and cyclopropyl PLFAs; arbuscular mycorrhizal fungi (AMF) use 16:1ω5c as representative marker; saprotrophic fungi (SF) use 18:1ω9c, 18:2ω6c and 18:3ω6c as representative markers^28–30^. The 12:0, 14:0, 15:0, 16:0, 17:0, 18:0 PLFAs were general markers present in all microorganisms^30,31^. Bacterial PLFAs included G+ and G− bacteria PLFAs. Fungal PLFAs included arbuscular mycorrhizal and saprotrophic fungi PLFAs. Total microbial PLFAs were the sum of bacterial, fungal, and general PLFAs.

Soil microbial biomass carbon (MBC), nitrogen (MBN), and phosphorus (MBP) were measured using the chloroform-extraction method^32,33^. For MBC and MBN, two fresh soil samples were used for the analysis. One sample was placed in a chloroform steam bath for 24 h and another sample was kept non-fumigated. Then, organic C and total N were extracted by shaking two soil samples in 0.5 M K_2_SO_4_ for 1 h and filtering through a Whatman No. 1 filter paper (9 cm in diameter). The filtered extracts were measured with a total organic carbon (TOC) analyzer (Elementar vario TOC, Hanau, Germany). Microbial biomass P was measured using a similar method as for MBC and MBN except that P was extracted by 0.5 M NaHCO_3_ and then measured with a UV Spectrometer (6505 spectrometer, Jenway, Stone, UK).

### DNA extraction and sequencing

We mixed ten soil samples of each plot to form one composite sample for DNA extraction and sequencing. Total genomic DNA was extracted from 0.5 g soil using a FastDNA Spin kit (MP Biomedical, Santa Ana, California, USA). The DNA quality was checked by 1% agarose gel electrophoresis and quantity was determined with a NanoDrop 2000 UV-vis spectrophotometer (Thermo Scientific, Wilmington). Bacterial 16 S rRNA genes were amplified with PCR primers 338 F (5′- ACTCCTACGGGAGGCAGCAG-3′) and 806 R (5′-GGACTACHVGGGTWTCTAAT-3′). Fungal internal transcribed spacer (ITS) rRNA genes were amplified with PCR primers ITS1F (5′-CTTGGTCATTTAGAGGAAGTAA-3′) and ITS2 (5′-GCTGCGTTCTTCATCGATGC-3′)^34,35^. The resulting PCR products were extracted from a 2% agarose gel and further purified using the AxyPrep DNA Gel Extraction Kit (Axygen Biosciences, Union City, CA, USA) and quantified using QuantiFluor™-ST (Promega, USA). Purified amplicons were pooled in equimolar concentrations and paired-end sequenced for high-throughput 16 S rRNA or ITS rRNA gene sequencing on an Illumina Hiseq. 2500 platform (Illumina Inc., USA) according to the standard protocols by Novogene Technology Co., Ltd. Operational taxonomic units (OTUs) were clustered with 97% similarity cut-off using UPARSE (version 7.1 https://drive5.com/uparse/), and chimeric sequences were identified and removed using UCHIME. Silva and Unite databases were used as references for bacteria and fungi, respectively^34,35^.

## Data Records

The dataset of plant and microbial community structure after long-term grazing and mowing in a semiarid steppe can be found in the Dryad repository^36^.

In the first dataset, each of the rows represents a quadrat, and the columns are defined as follows:

1. Block: Flat and sloped blocks.
2. MR: Management regime (grazing and mowing treatment).
3. GI: Grazing intensity (0, 1.5, 3.0, 4.5, 6.0, 7.5, 9.0 sheep ha^−1^).
4. Quadrat: Quadrat number (1, 2, 3, 4, 5, 6, 7, 8, 9, 10).
  5–99: Plant species: Plant species abundance (individual number).
  100. SR: Plant species richness in a quadrat.
  101. SD: Plant Shannon-Wiener diversity index in a quadrat.
  102. PE: Plant Pielou’s evenness in a quadrat.
  103. Coverage: Plant canopy cover (%).
  104. Height: Plant average height in a quadrat (cm).
  105. Moisture: Soil moisture (%).
  106. pH: Soil pH value.
  107. Hardness: Soil hardness (kg cm^−2^).
  108. SOC: Soil organic carbon (g kg^−1^).
  109. TN: Soil total nitrogen (g kg^−1^).
  110. TP: Soil total phosphorus (g kg^−1^).
  111. MBC: Microbial biomass carbon (mg kg^−1^).
  112. MBN: Microbial biomass nitrogen (mg kg^−1^).
  113. MBP: Microbial biomass phosphorus (mg kg^−1^).
  114. G+: G+ bacteria phospholipid fatty acids (nmol g^−1^ dry soil).
  115. G−: G− bacteria phospholipid fatty acids (nmol g^−1^ dry soil).
  116. Ba: Bacteria phospholipid fatty acids (nmol g^−1^ dry soil).
  117. SF: Saprotrophic fungi phospholipid fatty acids (nmol g^−1^ dry soil).
  118. AMF: Arbuscular mycorrhizal fungi phospholipid fatty acids (nmol g^−1^ dry soil).
  119. Fu: Fungi phospholipid fatty acids (nmol g^−1^ dry soil).
  120. Total: Total phospholipid fatty acids (nmol g^−1^ dry soil).
  121. G+/G−: Gram-positive bacteria to Gram-negative bacteria ratio.
  122. F/B: Fungi to Bacteria ratio.
  In the second dataset, we provide raw data for plant height. The columns are defined as follows:
  1–4. same as the first dataset.
5. Species: Plant species in each quadrat.
6. Avg.Height: Average height of each species (cm).

7–11. Height1, Height2, Height3, Height4, Height5: Plant height of 1^st^, 2^nd^, 3^rd^, 4^th^, and 5^th^ individual of each species (cm).

In the third and fourth datasets, we provide molecular sequencing data for bacteria and fungi, respectively. Both datasets have eight sheets with different levels of classification, i.e., Kingdom, Phylum, Class, Order, Family, Genus, Species, and OTUs. The first three columns have the same names with the first dataset. All other columns are bacteria and fungi names with relative abundance.

In the fifth dataset, we provide GPS coordinates and raw PLFA data for each quadrat. The first four columns have the same names as the first dataset. The fifth and sixth columns contain the longitude and latitude of each quadrat. All other columns are raw PLFA data with nmol g^−1^ dry soil for each PLFA marker.

## Technical Validation

**Fig. 2:**
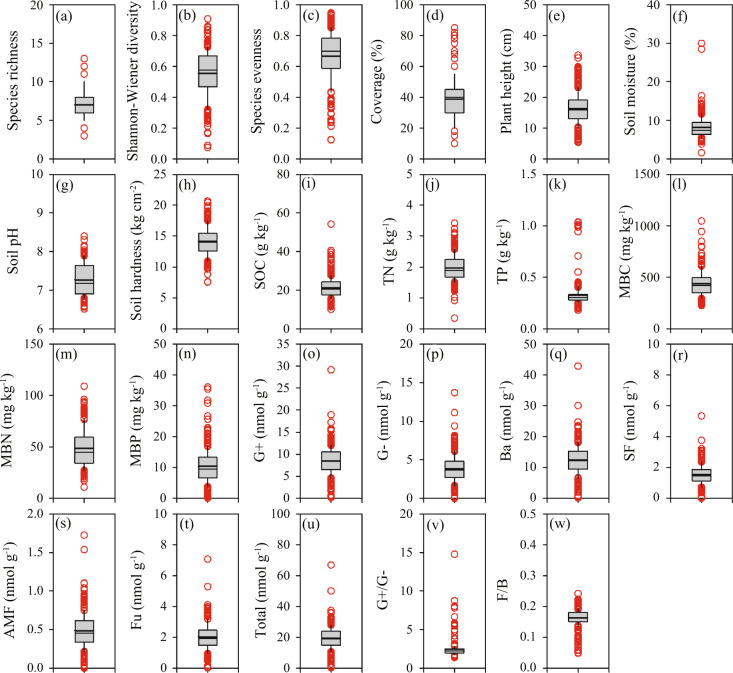
Boxplots of (**a**) species richness, (**b**) Shannon-Weiner diversity, (**c**) species evenness, (**d**) coverage, (**e**) plant height, (**f**) soil moisture, (**g**) soil pH, (**h**) soil hardness, (**i**) soil organic carbon, (**j**) soil total nitrogen, (**k**) soil total phosphorus, (**l**) microbial biomass carbon, (**m**) microbial biomass nitrogen, (**n**) microbial biomass phosphorus, (**o**) G+ bacteria, (**p**) G− bacteria, (**q**) bacteria, (**r**) saprotrophic fungi, (**s**) arbuscular mycorrhizal fungi, (**t**) fungi, (**u**) total phospholipid fatty acids, (**v**) G+/G− ratio, (**w**) F/B ratio. Mean, median, 25% and 75% percentiles, and outliers are shown.

All plant surveys were finished within two weeks to ensure data comparability. All soil samples were collected during the plant surveys and analysis was finished within one month of collection. We used boxplots to visualize data range and data distribution (Fig. 2). Meanwhile, we provided four potential explanations for the observed data range: (i) different topography, i.e., flat block vs. sloped block; the flat block has significant higher SOC and TN than sloped block at the beginning of the experiment (Table 1); (ii) different management regime, i.e., grazing vs. mowing, grazing is heterogeneous and returns nutrients to the soil, whereas mowing is homogeneous and removes nutrients from the soil; (iii) different grazing intensity, i.e., 0–9.0 sheep ha^−1^, both vegetation and soil properties could have a hump-shaped or linear decreased relationships with increasing grazing intensity^3^. (iv) grazing patches, i.e., lightly grazed vs. heavily grazed patches, selective grazing of livestock creates lightly and heavily grazed vegetation patches in the same plot. For example, there are two high soil moisture outliers in Fig. 2f. We suggest that others use the average values of other quadrats in the same plot instead of these outliers.

## Usage Notes

We encourage the use of the coefficient of variation (CV) to answer particular questions. For example, sheep grazing is selective and will result in heterogeneous patches in the subplots, on the contrary, mowing is not selective and will result in homogeneous community in the subplots. The CV of measured variables could reflect the effects of different management regimes on plant and microbial communities.
